# hENT1’s role in adjuvant intra-arterial gemcitabine-based chemotherapy for resectable pancreatic cancer patients

**DOI:** 10.1186/s12876-023-02666-x

**Published:** 2023-02-08

**Authors:** Wei Wang, Xinzhe Yu, Hengchao Li, Chuanxin Yang, Chen Jin, Xinyu Huang

**Affiliations:** 1 Department of Hepatobiliary and Pancreatic Surgery, Shanghai Sixth People’s Hospital Affiliated to Shanghai Jiao Tong University School of Medicine, 600 S. Yishan Road, Shanghai, 200233 China; 2 Department of Pancreatic Surgery, Huashan Hospital, Fudan University, 12 S. Middle Urumqi Road, Shanghai, 200040 China

**Keywords:** Gemcitabine-based regimen, Pancreatic cancer, hENT1, Prognostic marker, Intra-arterial infusion

## Abstract

**Background:**

We aimed to verify the role of hENT1 as a prognostic predictor for patients with resectable pancreatic ductal adenocarcinoma (PDAC) who underwent radical resection followed by intra-arterial infusion of gemcitabine-based regimen.

**Methods:**

We collected surgical samples from 102 patients with resectable PDAC who received radical resection followed by intra-arterial infusion of gemcitabine-based regimen. The hENT1 expression with the help of immunohistochemistry was conducted using formalin-fixed and paraffin embedded tissues. The Kaplan–Meier analyses and Cox regression were used to evaluate the mortality hazard associated with the discrepancy between strong and weak of hENT1 expression. Patients’ clinical and pathological characteristics were compared between the two groups, then the role of hENT1 as a prognostic predictor was further explored.

**Results:**

A total of 102 patients were included to assess the hENT1 expression. 50 patients were classified into high hENT1 expression group, the other 52 patients were attributed into low hENT1 expression group. High hENT1 expression was related to a significantly improved overall survival (OS) (*p* = 0.014) and disease-free survival (DFS) (*p* = 0.004). Both univariate (*p* = 0.001) and multivariate analyses (*p* < 0.001) indicated that high hENT1 expression was related to a decreased mortality.

**Conclusions:**

High expression of hENT1 is positive prognostic factor for adjuvant intra-arterial gemcitabine-based chemotherapy in resectable PDAC.

## Background

As the fourth leading cause of cancer-related deaths worldwide, pancreatic cancer has a dismal outcome with 5-year overall survival (OS) < 8% [1–3], despite the promising advances in basic and clinical researches. Although radical resection remains the only curative way to treat pancreatic cancer, there is still a high incidence rate of recurrence or metastases postoperatively [4]. The postoperative adjuvant chemotherapy becomes the standard therapeutic approach to reduce the recurrence or metastases after radical resection. Since the late 1990s, gemcitabine, a deoxycytidine analogue that inhibits DNA replication and repair, has become the first-line adjuvant chemotherapy for pancreatic cancer patients [5]. However, some patients are prone to recurrence and metastasis due to their resistance to gemcitabine. In addition, conventional way of using systematic chemotherapy may increase the risk of severe side effects. So, some researchers suggest regional intra-arterial infusion of chemotherapy (RIAIC) can be used as a new therapeutic strategy to deliver gemcitabine into the tumor tissues more selectively, which is a potential treatment for pancreatic cancer with increased regional therapeutic effects and decreased whole-body side effects [6–8]. The efficient biomarkers of regional chemotherapy sensitivity will contribute to select beneficiaries of this therapeutic strategy.

Because gemcitabine is strongly hydrophilic, it penetrates the hydrophobic cellular membrane slowly [9]. Therefore, the efficient endocytosis of gemcitabine urgently needs specialized membrane transporter proteins, among which human equilibrative nucleoside transporter 1 (hENT1) is a major one [10–12]. In vitro study suggested that hENT1 gene expression was positively associated with gemcitabine chemosensitivity [13]. Several clinical studies indicated that pancreatic cancer patients with high hENT1 expression in primary tumors have a longer survival after gemcitabine-based chemotherapy than patients with low hENT1 expression (14–17). However, other studies reported opposite results [18–20], in which they found that hENT1 levels did not predict prognosis in pancreatic cancer patients treated with gemcitabine-based chemotherapy. In addition, there is no report about the role of hENT1 expression on regional intra-arterial gemcitabine-based chemotherapy. So the role of hENT1 in gemcitabine chemosensitivity of pancreatic cancer needs to be verified, especially in regional intra-arterial infusion of chemotherapy.

In this study, we first verify the role of hENT1 as a prognostic predictor for pancreatic cancer patients who underwent radical surgery followed by intra-arterial infusion of gemcitabine-based chemotherapy, in order to select beneficiaries of this chemo-therapeutic strategy.

## Methods

### Clinicopathological data

From January 2015 to December 2018, 136 patients with radical resection of pancreatic cancer were retrospectively reviewed in the Shanghai Sixth People's Hospital and Huashan Hospital. The inclusion criteria: pancreatic ductal adenocarcinoma (PDAC) patients who had received radical surgery and postoperative intra-arterial infusion of chemotherapy; the diagnosis had been confirmed by postoperative pathology. The exclusion criteria: patients who had received preoperative adjuvant chemotherapy (n = 3) or other postoperative adjuvant therapy (n = 2), and those who had incomplete clinical data (n = 2), including one patient who was lost to follow up. Finally, a total of 102 patients were recruited into the analytical pool. All the clinicopathological characteristics were obtained from a periodically updated clinical database. The study was approved by the Ethics Committee of Shanghai Sixth People’s Hospital. Informed consent was obtained from all individual participants included in the study.

### Adjuvant chemotherapy

For the 102 patients, we performed a regional intra-arterial infusion to complete the postoperative chemotherapy. Briefly, chemotherapeutic agents were infused via a transfemorally placed catheter into the celiac axis and superior mesenteric artery. These patients received gemcitabine-based chemotherapy once every 28 days and treated three to five times. A single-drug regimen [gemcitabine (1000 mg/m^2^)] or a two-drug regimen [gemcitabine (1000 mg/m^2^) with oxaliplatin (85 mg/m^2^) or lobaplatin (50 mg/m^2^) or cisplatin (75 mg/m^2^)] was used. When the catheter came into the celiac axis, half of the dose were infused within 15 min. When the catheter came into the superior mesenteric artery, the other half of the dose were infused within 15 min.

### hENT1 immunohistochemistry

The primary tumor tissues from each patient were fixed by formalin and embedded by paraffin, then the immunohistochemistry (IHC) dyeing for hENT1 was conducted based on the standard protocol in previous reports [5, 21]. Concisely, the tissue slides de-paraffinized and autoclaved in citrate buffer at 95 °C for 40 min for unmasking antigens. The tissue slides were then incubated with a rabbit monoclonal hENT1 antibody (10D7G2, Abnova Co., Taipei, Taiwan) at 4 °C for 13 h, followed by dyeing with an avidin–biotin system (Shanghai High-tech Inc, Shanghai, China). All the nuclei were counterstained with hematoxylin.

The hENT1 expression was present within the islets of Langerhans cells and lymphocytes, which was used as internal references. Within in tumoral cells, hENT1 dyeing was major seen in the cytoplasm and cytomembrane area. The hENT1 dyeing analysis was assessed blindly by two experienced observers, and the final judgement was made by a third observer if there was any discrepancy. The hENT1 immunolabeling score was categorized as reported before [17]: the staining intensity for hENT1 expression was assigned a score from 0 to 3 based on 0+, no staining; 1+, weakly positive; 2+, moderately positive; and 3+, strongly positive. The percentage of positive tumor cells was scored as follows: 0+, no positive tumor cells; 1+, < 50% positive cells; 2+, 50–80% positive cells; and ≥ 81% positive cells. A composite score was obtained by calculating the sum of the above scores. The composite score ranges from 0 to 3 was assigned as low hENT1 expression and score ranges from 4 to 6 as high hENT1 expression.

### Follow up

The methods for follow-up included centers for disease control, outpatient service, electronic communication, etc. The OS and disease-free survival (DFS) were defined as the time period from operation to disease-specific death, and from operation to the development of either local recurrence or distant metastases, respectively. The expiry date of follow-up was December 30^th^, 2020.

### Statistical analysis

The Pearson's *X*^2^ test or Fisher’s exact probability test was applied to compare the clinicopathological characteristics of patients with high and low level of hENT1. For survival analysis, the Kaplan–Meier method, log-rank test and Cox regression analysis were used to assess the risk of mortality associated with the level of hENT1. All the statistical calculations were performed using SPSS software (version 23.0; IBM Inc., New York, NY, USA). A *P* < 0.05 was statistically significant.

## Results

### Patient’ clinical and pathological characteristics

**Fig. 1:**
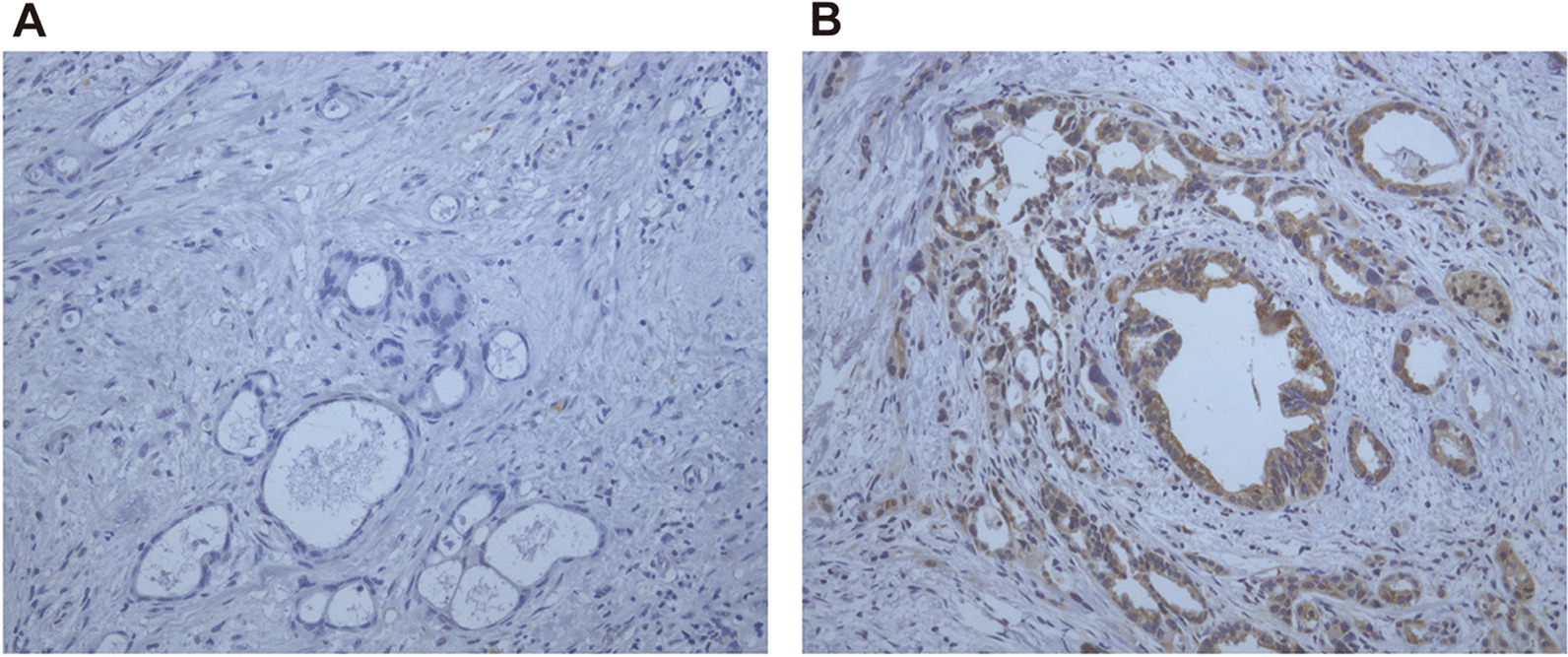
Immunohistochemical analysis of hENT1 expression in pancreatic ductal adenocarcinoma tissues. Representative immunohistochemical results of low hENT1 expression (**A**) and high hENT1 expression (**B**). Magnification × 200

The 102 patients consisted of 62 males and 40 females aged 44–82 years. Their detailed clinicopathological characteristics are presented in Table 1. They were divided into two groups according to the hENT1 expression level: the low hENT1 expression group (*n* = 52, 51%) (Fig. 1A) and the high hENT1 expression group (*n* = 50, 49%) (Fig. 1B). No significant differences in the basic data and risk factors including age, gender, tumor size, nodal status, tumor location, resection margin and dosing regimen were observed between the low hENT1 expression group and high hENT1 expression group.

**Table 1:** Clinicopathologic characteristics of 102 patients with pancreatic ductal adenocarcinoma

Characteristics	Low hENT1 expression group		High hENT1 expression group		*p* value^†^
	(n = 52)		(n = 50)		
	No	%	No	%	
Age					0.29^‡^
Mean ± SD	62.19 ± 8.93		64.32 ± 8.15		
Gender					0.33
Male	34	65.4%	28	56.0%	
Female	18	34.6%	22	44.0%	
Karnofsky performance status Score					1.00^‡^
Mean ± SD	75 ± 10		75 ± 10		
Tumor size					0.15
≥ 3 cm	40	76.9%	32	64.0%	
< 3 cm	12	23.1%	18	36.0%	
Nodal status					0.86
N0	30	57.7%	28	56.0%	
N1	22	42.3%	22	44.0%	
Tumor location					0.17
Head	36	69.2%	28	56.0%	
Body/tail	16	30.8%	22	44.0%	
Resection margin					0.47
R0	34	65.4%	36	72.0%	
R1	18	34.6%	14	28.0%	
DM					0.16
With	14	26.9%	20	40.0%	
Without	38	73.1%	30	60.0%	
Jaundice					0.17
With	16	30.8%	22	44.0%	
Without	36	69.2%	28	56.0%	
Dosing regimen					0.33
Single-drug regimen	31	59.6%	25	50.0%	
Two-drug regimen	21	40.4%	25	50.0%	

SD, standard deviation; DM, diabetes mellitus; ^†^χ^2^ test, except; ^‡^t test

### Role of hENT1 as a prognostic predictor

**Fig. 2:**
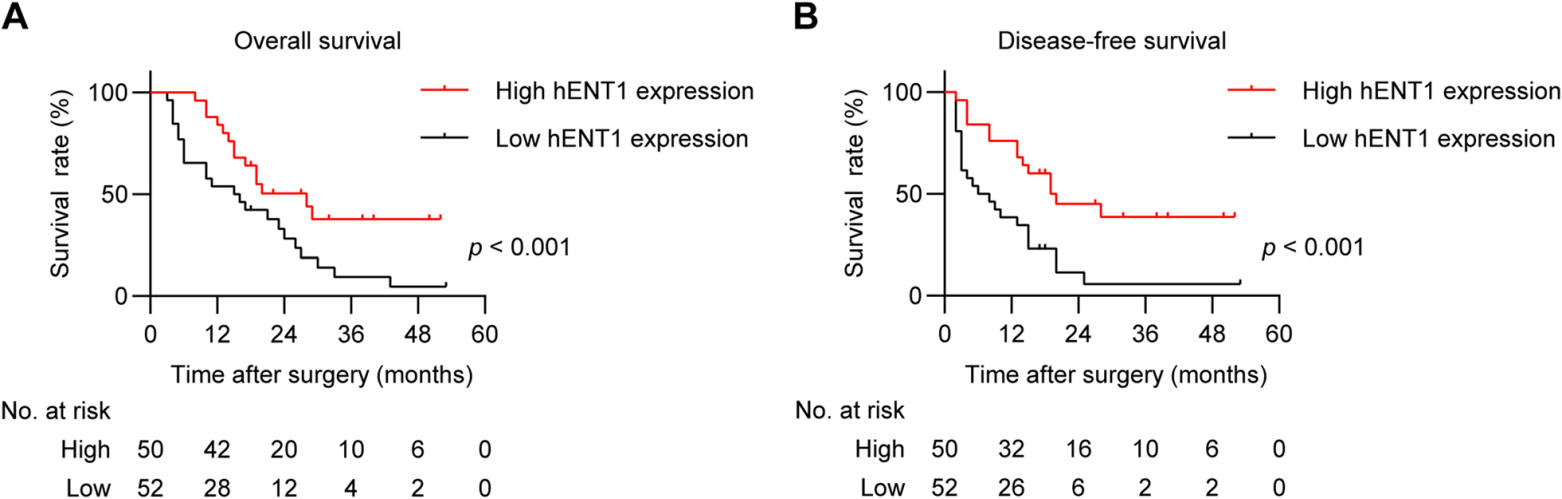
Kaplan–Meier survival curves of 102 patients with pancreatic ductal adenocarcinoma. **A** Patients with low hENT1 expression had significantly shorter median overall survival (OS) than those with high hENT1 expression (15 mon vs 28 mon, *P* < 0.001). **B** Patients with low hENT1 expression had significantly shorter median disease-free survival (DFS) than those with high hENT1 expression (6 mon vs 19 mon, *P* < 0.001)

102 patients were all followed (100%) with a median follow-up time of 30 months. Generally, all 102 patients had a median OS of 19 months and DFS of 14 months; the high hENT1 expression group had a median OS of 28 months and a median DFS of 19 months while the low hENT1 expression group had a median OS of 15 months and a median DFS of 6 months. Figure 2 demonstrated that high hENT1 expression in tumor cells was associated with significantly prolonged OS (*p* < 0.001) and DFS (*p* < 0.001). Notably, although the number of patients in the high hENT1 expression group was less than the other group (50 vs 52) initially, for every survey point after surgery, there were more patients alive in the high hENT1 expression group.

### Univariate and multivariate Cox regression analyses of overall survival

Univariate Cox regression analysis revealed that the following parameters were associated with increased mortality: low hENT1 expression in the primary tumor (HR 2.27; 95% CI 1.42–3.64; *p* = 0.001), positive lymph node (HR 1.78; 95% CI 1.12–2.83; *p* = 0.015), and poor tumor differentiation (HR 2.50; 95% CI 1.48–4.24; *p* = 0.001). The multivariate Cox regression analysis showed that low hENT1 expression in the primary tumor (HR 2.14; 95% CI 1.33–3.49; *p* = 0.002), positive lymph node (HR 1.97; 95% CI 1.23–3.16; *p* = 0.005), and poor tumor differentiation (HR 2.58; 95% CI 1.47–4.51; *p* = 0.001) as independent prognostic factors still retained their negative impact on survival (Table 2).

**Table 2:** Univariate and multivariate Cox regression analyses of factors affecting prognosis of 102 patients with pancreatic ductal adenocarcinoma after surgery

	Univariable Cox regression analysis		Multivariable Cox regression analysis	
	Hazard ratio (95%CI)	*p* value	Hazard ratio (95%CI)	*p* value
hENT1 expression in primary tumor				
Low versus high	2.27 (1.42–3.64)	0.001*	2.14 (1.33–3.49)	0.002*
Tumor size				
≥ 3 cm versus < 3 cm	1.20 (0.72–2.00)	0.496		
Nodal status				
N1 versus N0	1.78 (1.12–2.83)	0.015*	1.97 (1.23–3.16)	0.005*
Tumor differentiation				
Poorly versus well to moderately	2.50 (1.48–4.24)	0.001*	2.58 (1.47–4.51)	0.001*
Resection margin				
R1 versus R0	1.43 (0.87–2.33)	0.157		
Diabetes mellitus				
With versus without	1.00 (0.60–1.65)	0.983		
Jaundice				
With versus without	1.02 (0.63–1.63)	0.942		

*Significant difference

## Discussion

Gemcitabine must be transported across the plasma membrane as the first step to its conversion into active drugs, but it is strongly hydrophilic and associated with slow passive diffusion through hydrophobic cellular membranes. Therefore, the membrane permeability of tumor cells should be an important indicator to predict the efficacy of gemcitabine. Compared to systematic chemotherapy, this permeability may be more predictive in regional perfusion chemotherapy, because regional perfusion chemotherapy avoids systemic metabolism and directly acts in the local region. Efficient permeation of gemcitabine across cell membranes requires specialized integral membrane transporter proteins. Among these transporters, the hENT1 is the major mediator of gemcitabine uptake into human cells [22–26]. Cells lacking hENT1 are highly resistant to gemcitabine [26]. The hENT1 protein, which localizes in plasma and mitochondrial membranes, mediates the majority of gemcitabine transport in preclinical models [27–29]. The nucleoside transport inhibitors nitrobenzyl thioinosine or dipyridamole reduced sensitivity to gemcitabine by 39- to 1800-fold [26]. These data of basic research laid the foundation for hENT1 to become a biomarker for the chemotherapy sensitivity of gemcitabine, which needs to be further confirmed by clinical studies especially in regional intra-arterial infusion chemotherapy.

Most clinical studies including systematic reviews and meta-analyses reached the conclusion that high hENT1 expression is predictive of gemcitabine sensitivity in the systematic chemotherapy of PDACpatients [30–34]. Especially in adjuvant chemotherapy, a number of studies already reported the hENT1 is a strong independent prognostic factor in PDAC patients who receive gemcitabine-based chemotherapy after surgery. Compared to the above studies, our study first investigates the role of hENT1 expression in regional intra-arterial infusion of gemcitabine after radical resection in resectable PDACpatients. The results showed that high hENT1 expression in tumor cells is associated with a significantly increased OS and DFS. These data that high hENT1 expression in PDAC is an important sensitive marker of gemcitabine-based chemotherapy, including systematic and local chemotherapy.

Accurate methods and procedures of tumor sample detection are crucial to judge the predictive value of hENT1. Attention should be paid to the following aspects: (1) Selection of specific antibody. It has been reported that the expression level of hENT1 detected by the specific antibody used in our study has a strong predictive value, while other antibodies have poor specificity and predictive value [35]. (2) Selection of samples from targeted localizations. There are differences of hENT1 expression between samples from primary localizations and metastatic localizations, as well as between simultaneous and metachronous metastases [35]. (3) Selection of samples taken before chemotherapy. Studies were considered eligible if they met the criteria: measurement of pretreatment values and evaluation of the potential association between pretreatment hENT1 and the survival outcome. For example, a retrospective study reported hENT1 level did not predict prognosis in PDACpatients treated with neoadjuvant chemoradiation including gemcitabine, in which hENT1 expression was evaluated in the surgical sample after neoadjuvant chemoradiation [18]. This contradictory result might be explained by the potential preferential eradication of tumor cells with high expression of hENT1 by the neoadjuvant treatment. (4) Selection of IHC analysis. Although the predictive value of hENT1 expression measured by IHC are similar to polymerase chain reaction (PCR), IHC analysis is used widely for evaluating molecular markers in clinical tissue specimens [30, 31, 35]. Several more sophisticated methods, such as cDNA microarray, fluorescence in situ hybridization and quantitative reverse transcriptase PCRare still impractical in routine clinical settings. (5) Selection of surgically resected specimens. The predictive value of hENT1 expression level detected in needle biopsy specimens is inferior to the surgically resected specimens [36]. Thus, the studies of predictive value of hENT1 in palliative and neoadjuvant chemotherapy often draw negative conclusions, as needle biopsy samples are often used to detect hENT1 expression level [19, 20]. However, obtaining surgical resection specimen is convenient for the detection of hENT1 expression before postoperative adjuvant chemotherapy. Therefore, the conclusions of our study are reliable under the premise of following the above procedures. In our study, multivariate analyses showed that low hENT1 expression in the primary tumor is an independent prognostic factor for the regional intra-arterial infusion of gemcitabine in PDACpatients after radical resection.

## Conclusions

In summary, our study shows that the PDACpatients with high hENT1 expression may benefit from regional intra-arterial infusion chemotherapy of gemcitabine. hENT1 becomes an available biomarker for the chemotherapy sensitivity of gemcitabine in the treatment of PDACpatients, especially in postoperative regional intra-arterial infusion chemotherapy. Future investigation of hENT1's role in this type of treatment, if possible, could shed light on performing a randomized controlled trial.

## Data Availability

The data used and/or analysed during the current study are available from the corresponding author on reasonable request.

## Abbreviations

OS: Overall survival
RIAIC: Regional intra-arterial infusion of chemotherapy
hENT1: Human equilibrative nucleoside transporter 1
PDAC: Pancreatic ductal adenocarcinoma
DFS: Disease-free survival
HR: Hazard ratio
CI: Confidence interval
IHC: Immunohistochemistry

## References

[1] Siegel RL, Miller KD, Fuchs HE (2021). Cancer statistics, 2021. CA Cancer J Clin.

[2] Vaccaro V, Sperduti I, Milella M (2011). FOLFIRINOX versus gemcitabine for metastatic pancreatic cancer. N Engl J Med..

[3] Hidalgo M (2010). Pancreatic cancer. N Engl J Med.

[4] Greenhalf W, Ghaneh P, Neoptolemos JP (2014). Pancreatic cancer hENT1 expression and survival from gemcitabine in patients from the ESPAC-3 trial. J Natl Cancer Inst.

[5] Murata Y, Hamada T, Kishiwada M (2012). Human equilibrative nucleoside transporter 1 expression is a strong independent prognostic factor in UICC T3–T4 pancreatic cancer patients treated with preoperative gemcitabine-based chemoradiotherapy. J Hepatobiliary Pancreat Sci.

[6] Liu X, Yang X, Zhou G (2016). Gemcitabine-based regional intra-arterial infusion chemotherapy in patients with advanced pancreatic adenocarcinoma. Medicine (Baltimore).

[7] Ohigashi H, Ishikawa O, Imaoka S (1996). A new method of intra-arterial regional chemotherapy with more selective drug delivery for locally advanced pancreatic cancer. Hepatogastroenterology.

[8] Cantore M, Pederzoli P, Cornalba G (2000). Intra-arterial chemotherapy for unresectable pancreatic cancer. Ann Oncol.

[9] Marechal R, Bachet JB, Mackey JR (2012). Levels of gemcitabine transport and metabolism proteins predict survival times of patients treated with gemcitabine for pancreatic adenocarcinoma. Gastroenterology..

[10] Jiang HB, Xu M, Wang XP (2008). Pancreatic stellate cells promote proliferation and invasiveness of human pancreatic cancer cells via galectin-3. World J Gastroenterol WJG.

[11] Yamaue H, Tani M, Onishi H (2002). Locoregional chemotherapy for patients with pancreatic cancer intra-arterial adjuvant chemotherapy after pancreatectomy with portal vein resection. Pancreas.

[12] Omori H, Nio Y, Takeda H (1996). Application for therapeutic use of deuterium oxide (D2O) against human pancreatic cancer. Cancer Chemother.

[13] Mori R, Ishikawa T, Ichikawa Y (2007). Human equilibrative nucleoside transporter 1 is associated with the chemosensitivity of gemcitabine in human pancreatic adenocarcinoma and biliary tract carcinoma cells. Oncol Rep.

[14] Spratlin J, Sangha R, Glubrecht D (2004). The absence of human equilibrative nucleoside transporter 1 is associated with reduced survival in patients with gemcitabine-treated pancreas adenocarcinoma. Clin Cancer Res.

[15] Giovannetti E, Del Tacca M, Mey V (2006). Transcription analysis of human equilibrative nucleoside transporter-1 predicts survival in pancreas cancer patients treated with gemcitabine. Cancer Res.

[16] Farrell JJ, Elsaleh H, Garcia M (2009). Human equilibrative nucleoside transporter 1 levels predict response to gemcitabine in patients with pancreatic cancer. Gastroenterology.

[17] Morinaga S, Nakamura Y, Watanabe T (2012). Immunohistochemical analysis of human equilibrative nucleoside transporter-1 (hENT1) predicts survival in resected pancreatic cancer patients treated with adjuvant gemcitabine monotherapy. Ann Surg Oncol.

[18] Kawada N, Uehara H, Katayama K (2012). Human equilibrative nucleoside transporter 1 level does not predict prognosis in pancreatic cancer patients treated with neoadjuvant chemoradiation including gemcitabine. J Hepatobiliary Pancreat Sci.

[19] Poplin E, Wasan H, Rolfe L (2013). Randomized, multicenter, phase II study of CO-101 versus gemcitabine in patients with metastatic pancreatic ductal adenocarcinoma: including a prospective evaluation of the role of hENT1 in gemcitabine or CO-101 sensitivity. J Clin Oncol Off J Am Soc Clin Oncol.

[20] Ormanns S, Heinemann V, Raponi M (2014). Human equilibrative nucleoside transporter 1 is not predictive for gemcitabine efficacy in advanced pancreatic cancer: translational results from the AIO-PK0104 phase III study with the clone SP120 rabbit antibody. Eur J Cancer.

[21] Yu X-Z, Guo Z-Y, Di Y (2017). The relationship between SPARC expression in primary tumor and metastatic lymph node of resected pancreatic cancer patients and patients' survival. Hepatobiliary Pancreat Dis Int.

[22] Nakano Y, Tanno S, Koizumi K (2007). Gemcitabine chemoresistance and molecular markers associated with gemcitabine transport and metabolism in human pancreatic cancer cells. Br J Cancer.

[23] Michalski CW, Erkan M, Sauliunaite D (2008). Ex vivo chemosensitivity testing and gene expression profiling predict response towards adjuvant gemcitabine treatment in pancreatic cancer. Br J Cancer.

[24] Ohhashi S, Ohuchida K, Mizumoto K (2008). Down-regulation of deoxycytidine kinase enhances acquired resistance to gemcitabine in pancreatic cancer. Anticancer Res.

[25] Zhang J, Visser F, King KM (2007). The role of nucleoside transporters in cancer chemotherapy with nucleoside drugs. Cancer Metastasis Rev.

[26] Damaraju VL, Damaraju S, Young JD (2003). Nucleoside anticancer drugs: the role of nucleoside transporters in resistance to cancer chemotherapy. Oncogene.

[27] Mackey JR, Mani RS, Selner M (1998). Functional nucleoside transporters are required for gemcitabine influx and manifestation of toxicity in cancer cell lines. Cancer Res.

[28] Ritzel MW, Ng AM, Yao SY (2001). Molecular identification and characterization of novel human and mouse concentrative Na+-nucleoside cotransporter proteins (hCNT3 and mCNT3) broadly selective for purine and pyrimidine nucleosides (system cib). J Biol Chem.

[29] Garcia-Manteiga J, Molina-Arcas M, Casado FJ (2003). Nucleoside transporter profiles in human pancreatic cancer cells: role of hCNT1 in 2',2'-difluorodeoxycytidine- induced cytotoxicity. Clin Cancer Res.

[30] Liu ZQ, Han YC, Zhang X (2014). Prognostic value of human equilibrative nucleoside transporter1 in pancreatic cancer receiving gemcitabin-based chemotherapy: a meta-analysis. PLoS ONE.

[31] Ansari D, Rosendahl A, Elebro J (2011). Systematic review of immunohistochemical biomarkers to identify prognostic subgroups of patients with pancreatic cancer. Br J Surg.

[32] Nordh S, Ansari D, Andersson R (2014). hENT1 expression is predictive of gemcitabine outcome in pancreatic cancer: a systematic review. World J Gastroenterol: WJG.

[33] Bird NT, Elmasry M, Jones R (2017). Immunohistochemical hENT1 expression as a prognostic biomarker in patients with resected pancreatic ductal adenocarcinoma undergoing adjuvant gemcitabine-based chemotherapy. Br J Surg.

[34] Perera S, Jang GH, Wang Y (2022). hENT1 expression predicts response to gemcitabine and nab-paclitaxel in advanced pancreatic ductal adenocarcinoma. Clin Cancer Res.

[35] Raffenne J, Nicolle R, Puleo F (2019). hENT1 testing in pancreatic ductal adenocarcinoma: are we ready? A multimodal evaluation of hENT1 status. Cancers (Basel)..

[36] Yabushita Y, Mori R, Taniguchi K (2017). Combined analyses of hENT1, TS, and DPD predict outcomes of borderline-resectable pancreatic cancer. Anticancer Res.

